# Acid sensing ion channel 2: A new potential player in the pathophysiology of multiple sclerosis

**DOI:** 10.1111/ejn.14302

**Published:** 2019-02-19

**Authors:** Teresa Fazia, Roberta Pastorino, Serena Notartomaso, Carla Busceti, Tiziana Imbriglio, Milena Cannella, Davide Gentilini, Gabriele Morani, Anna Ticca, Pierpaolo Bitti, Carlo Berzuini, Tamas Dalmay, Giuseppe Battaglia, Luisa Bernardinelli

**Affiliations:** ^1^ Department of Brain and Behavioral Sciences University of Pavia Pavia Italy; ^2^ I.R.C.C.S. Neuromed Pozzilli Italy; ^3^ Department of Physiology and Pharmacology University Sapienza Roma Italy; ^4^ Unità di Bioinformatica e Statistica Genomica Istituto Auxologico Italiano IRCCS Cusano Milanino Milano Italy; ^5^ Azienda Tutela Salute Sardegna ASSL Nuoro Neurologia e Stroke Unit Ospedale “San Francesco” Nuoro Italy; ^6^ Azienda Tutela Salute Sardegna ASSL Nuoro Immunoematologia e Medicina Trasfusionale Ospedale “San Francesco” Nuoro Italy; ^7^ Centre for Biostatistics University of Manchester Manchester UK; ^8^ School of Biological Sciences University of East Anglia Norwich UK

**Keywords:** ASIC1, ASIC2, experimental autoimmune encephalomyelitis, mechanosensation, mouse models

## Abstract

Acid‐sensing ion channels (ASICs) are proton‐gated channels involved in multiple biological functions such as: pain modulation, mechanosensation, neurotransmission, and neurodegeneration. Earlier, we described the genetic association, within the Nuoro population, between Multiple Sclerosis (MS) and rs28936, located in ASIC2 3′UTR. Here we investigated the potential involvement of ASIC2 in MS inflammatory process. We induced experimental autoimmune encephalomyelitis (EAE) in wild‐type (WT), knockout Asic1^−/−^ and Asic2^−/−^ mice and observed a significant reduction of clinical score in Asic1^−/−^ mice and a significant reduction in the clinical score in Asic2^−/−^ mice in a limited time window (i.e., at days 20–23 after immunization). Immunohistochemistry confirmed the reduction in adaptive immune cell infiltrates in the spinal cord of EAE Asic1^−/−^ mice. Analysis of mechanical allodynia, showed a significant higher pain threshold in Asic2^−/−^ mice under physiological conditions, before immunization, as compared to WT mice and Asic1^−/−^. A significant reduction in pain threshold was observed in all three strains of mice after immunization. More importantly, analysis of human autoptic brain tissue in MS and control samples showed an increase of ASIC2 mRNA in MS samples. Subsequently, *in vitro* luciferase reporter gene assays, showed that ASIC2 expression is under possible miRNA regulation, in a rs28936 allele‐specific manner. Taken together, these findings suggest a potential role of ASIC2 in the pathophysiology of MS.

**Abbreviations**
ASIC: Acid sensing ion channel
CI: Confidence Interval
CNS: Central nervous system
E&H: Hematoxylin and eosin
EAE: Experimental autoimmune encephalomyelitis
MDEG: Mammalian degenerin
MHC: Major Histocompatibility Complex
MS: Multiple Sclerosis
NGS: Next Generation Sequencing
PPMS: Primary Progressive Multiple Sclerosis
SNP: Single nucleotide polymorphism
WT: Wild‐type

## 1 INTRODUCTION

Over the last decade, genetic research on multiple sclerosis (MS) has led to the discovery of more than 100 non‐MHC (Major Histocompatibility Complex) susceptibility variants, which play an important role in both the innate and the adaptive immune system, with a prominent representation of the latter (Beecham et al., 2013; Consortium et al., 2011; Dendrou, Fugger, & Friese, 2015; Lill et al., 2015; Moutsianas et al., 2015; Patsopoulos et al., 2011). Genes driving the Central Nervous System's (CNS) intrinsic inflammatory arm still deserve close examination in order to clarify the mechanisms of axonal degeneration in MS and the genetic contributions to disease severity or subphenotypes (Hoppenbrouwers & Hintzen, 2011).

We focused our study on ASIC2, an interesting candidate gene widely expressed in the brain.

The idea of focusing on this gene came from the discovery, during our previous work on the isolated population of the Nuoro province (Sardinia, Italy), of a significant association between MS and (a) the microsatellite D17S798 in the 17q11.2 region, a marker close to ASIC2 and (b) the 3′UTR single nucleotide polymorphism (SNP) rs28936 (Bernardinelli et al., 2007). Additional interest on ASIC2 was provided by a genetic study on mice with experimental autoimmune encephalomyelitis (EAE), a mouse model of MS (Jagodic et al., 2004), where strong evidence of linkage in correspondence of the Eae18b QTL was found; in particular, ASIC2 is the closest gene to the peak linkage marker D10Rat123.

ASIC2 encodes the mammalian degenerin (MDEG) protein and in the nematode *Caenorhabditis Elegans* mutations of MDEG homologues known as degenerins (deg‐1, mec‐4, mec‐10) are involved in neurodegeneration (Waldmann, Champigny, Voilley, Lauritzen, & Lazdunski, 1996). ASIC2 is a member of the ASIC (acid sensing ion channel) family. From four different genes (ASIC1, ASIC2, ASIC3, and ASIC4), 6 subunits have been reported so far: ASIC1a, ASIC1b, ASIC2a, ASIC2b, ASIC3, and ASIC4 to form proton‐dependent ion channels that are mainly permeable to sodium. Among them, ASIC1a and ASIC2a are highly expressed in the CNS, where they interact to form functional homotrimeric or heterotrimeric complexes, with variable stoichiometric ratios (2:1 or 1:2), different sensitivity to pH, and unique biophysical properties (Bartoi, Augustinowski, Polleichtner, Gründer, & Ulbrich, 2014; Wu, Xu, et al., 2016). ASIC2a is less sensitive to pH changes with respect to ASIC1a, ASIC1b and ASIC3, although its sensitivity changes when it heteromultimerizes with other subunits. ASIC1a homomers and ASIC1a‐ASIC2b heteromers also show, in addition to Na^+^, a permeability for Ca^2+^ (Ortega‐Ramírez, Vega, & Soto, 2017) and an excessive accumulation of these ions is known to be involved in neurodegeration and inflammation in MS (Friese et al., 2007). ASIC1a is the key subunit determining acid‐activated current in CNS, thus playing a critical role in neurological and psychological diseases; while ASIC2a play important modulatory roles in acid‐induced responses (Wu, Leng, et al., 2016). ASIC2b is not sensitive to acid and does not form functional homomeric channels, but it contributes to proton‐sensing by forming heteromeric channels with other ASIC subunits (Zhou et al., 2016). In mice, ASIC1 and ASIC2 subunits often colocalize in multiple brain regions, despite the differences observed in certain brain areas (Price et al., 2014).

Our interest in this class of genes, and in particular on ASIC2, was further motivated by literature evidence showing that ASIC channels are involved in multiple biological functions such as: pain modulation, mechanosensation, acidosis‐induced neuronal death and neurotransmission (Kang et al., 2012; Kweon & Suh, 2013; Ortega‐Ramírez et al., 2017; Sherwood, Lee, Gormley, & Askwith, 2011; Wemmie, Taugher, & Kreple, 2013). ASICs are activated in response to extracellular acidification occurring in neurotransmission at synaptic level (reviewed in Boscardin, Alijevic, Hummler, Frateschi, & Kellenberger, 2016). Most recently, ASICs have been involved in regulating synaptic plasticity in the amygdala (Du et al., 2014) and the generation of postsynaptic currents by ASIC1a and ASIC2 was reported to act on the reduction of cocaine addiction (Kreple et al., 2014). They also play an important role in detecting the local pH changes induced by inflammation‐associated processes (Radu et al., 2014; Rajamäki et al., 2013). Importantly, ASICs have been linked to immunological function, due to their ability of enhancing antigen presentation of dendritic cells (Tong et al., 2011). Evidence of ASICs involvement in axonal degeneration firstly came from a study performed in EAE mice carrying two copies of a genetically inactivated ASIC1 gene (Asic1^−/−^) (Friese et al., 2007). This study also showed that a hypoxia‐induced dysfunction in mitochondrial metabolism evokes acidosis in the neuronal tissue of the EAE‐induced mice, which activates ASIC1. Subsequent studies performed on both acute and chronic EAE models demonstrated that ASIC1 upregulation and function play an important role in the axonal and myelin damage in acute and chronic‐relapsing EAE, while blocking ASIC1 with amiloride exerted a neuro‐ and also myelo‐protective effect in both acute and chronic EAE (Vergo et al., 2011). In autoptic brain tissues from patients affected by Primary Progressive MS (PPMS) upregulation of ASIC1 was also observed, and amiloride administration improved tissue lesions in PPMS thus exerting a neuroprotective effect, as indicated in a pilot trial (Arun et al., 2013). Converging statistical and biological evidences implicate ASICs as putative MS susceptibility genes and the investigation of their role in MS has just begun (Zhou et al., 2016). Despite the fact that most studies cited above were focused on ASIC1, it is reasonable to hypothesize that ASIC2 could also play an important role in MS pathogenesis, as the two channels interact. Our research aimed at filling this gap by investigating the possible involvement of ASIC2 in MS inflammatory and neurodegenerative processes, via different experimental *in vivo* and *in vitro* approaches. A further aim was to replicate the results obtained for ASIC1 by a previous study in EAE mice (Friese et al., 2007) and to interpret them jointly with those obtained for ASIC2.

## 2 MATERIAL AND METHODS

### 2.1 Workflow of the study

The initial idea of this study came after finding evidence of a statistically significant association, obtained in our previous study on Sardinian families, between the ASIC2 3′UTR variant rs28936, located in a miRNA‐binding site, and MS.

Despite our research primarily is focuded on ASIC2, we also tried to replicate the experiments, mostly obtained by Friese et al. (2007), on EAE mice showing a strong involvement of ASIC1 gene on neurodegeneration.

This logic led us to plan the following experiments:

- Analyze clinical scores and pain thresholds in wild‐type (WT) and Asic2^−/−^ mice with induced EAE, in different phases of the disease;
- Perform histology and immunohistochemistry of cerebellum and spinal cord in WT and Asic2^−/−^ mice;
- Study ASIC2 gene expression on post‐mortem brain tissue of MS patients and controls;
- Investigate the role of rs28936 as a possible miRNA‐binding site.
- Perform the same experiment as (a) and (b) on Asic1^−/−^ mice for sake of replication of the published previous results.

### 2.2 ASICs involvement in EAE

All experimental procedures on animals were performed in accordance with the Italian (D.L. 26/2014) and European Union Directive (2010/63/EU) on the protection of animals used for scientific purposes. Experiments were approved by the Italian Ministry of Health. All efforts were made to minimize the number of animals used and to alleviate their discomfort.

Wild‐type, Asic1^−/−^ and Asic2^−/−^ male mice (kindly provided by Dr. Margaret Price, University of Iowa, Iowa City, IA, USA) at 6/7 weeks of age were used. All mice were obtained by homozygous breeding. Mice were kept under environmentally controlled conditions (ambient temperature, 22°C; humidity, 40%) on a 12‐hr light/dark cycle with food and water *ad libitum*. EAE, a T cell‐dependent mouse model of MS that recapitulates many of the clinical and neuropathological modifications observed in the human disease, was induced in WT (*n* = 14), Asic1^−/−^ (*n* = 10) and Asic2^−/−^ (*n* = 14) mice by subcutaneous (s.c.) immunization with 200 μg of MOG35‐55 peptide (MEVGWYRSPFSRVVHLYRNGK – Genemed Synthesis, San Antonio, TX, USA) emulsified in 0.1 ml of incomplete Freund's adjuvant (Sigma‐Aldrich, St. Louis, MO, USA) containing 2 mg of *Mycobacterium tuberculosis* strain H37Ra (Difco Laboratories, Detroit, MI, USA). 200 ng of pertussis toxin (Calbiochem, La Jolla, CA, USA) dissolved in 200 μl phosphate‐buffered saline were injected intraperitoneally on the day of immunization and 48 hrs later. Mice were weighted and monitored daily for 40 days and neurological signs were scored according to the following scale: 0 = no symptoms; 1 = limp tail; 2 = partial paralysis of hind limbs; 3 = complete paralysis of hind limbs or partial hind and front limb paralysis; 4 = tetraparalysis; 5 = moribund/death. Forty‐five days after MOG immunization, mice were killed and spinal cords were dissected out for histological and immunohistochemical examination.

The experiments were performed by an unbiased method carried out by expert experimenter unaware of the mouse genotype. Statistical analysis was performed by a Two‐way ANOVA Repeated Measures + Bonferroni post hoc test. To evaluate the existence of a statistically significant difference in the pattern of clinical score in WT vs Asic1^−/−^ and WT vs Asic2^−/−^ in the 40 days after immunization, we also calculated for each time point the difference in the mean of the observed clinical scores between WT and Asic1^−/−^ and WT and Asic2^−/−^. To perform a statistical test of the null hypothesis of no difference between the means of the mice groups being compared, we simulated 100,000 samples under the null hypothesis and we plotted the 90% non‐parametric confidence intervals (CIs) by identifying the interval including 90% of the samples, in the empirical distribution of the simulated values. This approach takes into account the correlation between clinical scores observed in the same mouse at different days after immunization. We computed a 90% CI (i.e., 5% one‐tail test) since our *a priori* expectation is that the lack of ASIC2/ASIC1 gene implicates an improvement of the clinical score in comparison to that observed in WT. We tested the hypothesis that the difference between Asic2^−/−^/Asic1^−/−^ and WT profiles is significantly different from zero and negative. Data points lying outside the lower confidence band can be interpreted as evidence of a departure from the null hypothesis.

### 2.3 Histology and immunohistochemistry analysis of spinal cord of EAE mice

Mice were killed by decapitation after administration of a light anesthesia with isoflurane and histological analysis and immunohistochemistry were performed as previously reported (Fallarino et al., 2010) on three mice for each experimental group representative of WT, Asic1^−/−^ and Asic2^−/−^ mice with induced EAE. Thirty μm sections of the spinal cord were stained by hematoxylin/eosin to reveal CNS inflammatory infiltrates. For immunohistochemical analysis, 30 μm sections were soaked in 3% hydrogen peroxide to block endogenous peroxidase activity and then stained with rat monoclonal anti‐CD4 (1:500; ab64144, Abcam, Cambridge, UK), mouse monoclonal anti‐class II MHC (1:500; MAB 2221, Chemicon, Tecumela, CA, USA) or rat monoclonal anti‐CD8 (1:100; NBP1‐49045, Novus Biologicals, Littleton, CO, USA) followed by appropriate biotinylated secondary antibodies (anti‐mouse, 1:200; BA2000 or anti‐rat, 1:200; BA4000, Vector Laboratories, Burlingame, CA, USA) and streptavidin‐HRP (Zymed, Waltham, MA, USA). For the quantification of the inflammation in the spinal cord white matter, the percentage of white matter infiltrated by CD4^+^, CD8^+^, and MHCII cells in transverse spinal cord sections of three EAE mice was determined by acquiring images at 10X and analyzing with the Image J software. Data were analyzed by using one‐way ANOVA + Fisher's least significant difference (LSD).

### 2.4 Assessment of mechanical allodynia in mice

Mechanical pain thresholds were quantified in WT (*n* = 20), Asic1^−/−^ (*n* = 12) and Asic2^−/−^ (*n* = 21) mice under basal conditions (prior immunization), and at 28 and 40 days after immunization, by measuring the hind paw withdrawal response to von Frey filament stimulation. Mice were placed in a Plexiglas box (20 cm high, 9 cm diameter) with a wire grid bottom through which the von Frey filaments (North Coast Medical, Inc., San Jose, CA, USA), bending force range from 0.008 to 3.5 g, were applied by using a modified version of the up‐down paradigm, as previously reported (Chaplan, Bach, Pogrel, Chung, & Yaksh, 1994). The filaments were applied five times each and pressed perpendicularly to the plantar surface of both right‐ and left‐hind paws until they bent. The first filament, which evoked at least three responses, was assigned blindly as the pain threshold in grams. The experiments were performed by an unbiased method carried out by expert experimenter unaware of the mouse genotype. Data were analyzed by using one‐way ANOVA + Fisher's LSD.

### 2.5 Quantification of ASIC2 expression in autoptic human brain tissues

ASIC2 expression was quantified in 62 cases with secondary progressive MS and 32 controls (affected by other non‐neurological diseases) in brain tissues from different brain regions including occipital, frontal, parietal and temporal lobes, cerebellum, thalamus and hippocampus. Human tissue samples were kindly provided by the Multiple Sclerosis Society Tissue Bank of the Imperial College, London (Burlington Danes, 160 Du Cane Road, London W12; www.ukmstissuebank.imperial.ac.uk). For four samples, one of the two measurements were missing; we imputed these missing values via multivariate imputation by chained equation (MICE R package; van Buuren & Groothuis‐Oudshoorn, 2011). The whole 3′UTR region (chr17:31,340,123‐31,340,982 – GRCh37/hg19) containing the marker rs28936 was fully genotyped by Next Generation Sequencing (NGS). Briefly, the region of interest was amplified in each sample by PCR reaction, using the couple of primers (Forward – TCTTAACCTGCCCAAAAACC and Reverse – GGGAGAGAAGAACGACATGG). PCR reaction was performed in a total volume of 25 μl containing 50 ng genomic DNA, 5 pmol of each primer, 1 × Taq polymerase buffer (1.5 mm MgCl_2_) and 0.5 units of Red Taq (Sigma).

The PCR amplification was carried out for 35 cycles (denaturation at 94°C for 45 s, annealing for 1 min at 55°C, extension at 72°C for 45 s and final extension for 10 min at 72°C). Libraries for NGS analysis were obtained from PCR amplicons by NGS Illumina NEXTERA XT assay and following the procedures indicated by the vendor. Libraries were finally analyzed with Illumina Miseq sequencer. Bioinformatics analysis was performed by the software Miseq reporter in order to obtain for each sample analysed a list of Genomic variants in a VCF format file. Genotyping data about rs28936 have been extracted from VCF files and recorded in a pedigree format files.

Total RNA from human brain samples was extracted using Trizol reagent (Invitrogen, Carlsbad, CA, USA) according to manufacturer's protocol. The RNA was treated with DNAse (Qiagen, Hilden, Germany) and single strand cDNA was synthesized from 2 μg of total RNA using superscript III (Invitrogen) and random hexamers. Real‐time PCR was performed on 20 ng of cDNA by using specific primers and Power SYBR Green Master Mix (Applied Biosystem, Foster City, CA) on an Applied Biosystems Step‐One instrument. Thermal cycler conditions were: 10 min at 95°C, 40 cycles of denaturation (15 s at 95°C), and combined annealing/extension (1 min at 60°C).

Primers used were as follows: ASIC2 Forward TGAGTGCCGATCCTCAGAGA and Reverse TGTAGCGGGTTAGGTTGCAG and GAPDH Forward TTGCCATCAATGACCCCTTCA and Reverse CGCCCCACTTGATTTTGGA. Here, mRNA copy number of ASIC2 gene was calculated from serially diluted standard curves simultaneously amplified with the samples and normalized against GAPDH copy number. Two technical replicates were run for each PCR reaction. *P* < 0.05 at Student's *t* test was considered significant.

### 2.6 Luciferase reporter gene assay

In order to investigate whether ASIC2 expression is under miRNAs regulation dependent on rs28936 genotype, we performed a luciferase reporter gene assay. ASIC2 3′UTR was cloned from two different genotypes (rs28936A and rs28936G) downstream of the firefly luciferase gene in the dual‐luciferase plasmid pmirGLO (Promega) and the two constructs were sequenced to confirm the correct insertion of the fragments. We transfected both constructs into MCF7 cells (TCC‐HTB‐22) in the presence or absence of a siRNA that targets Dicer or a non‐specific siRNA (negative control). The cells were cultured in a 96‐well plate starting at a density of 4 × 10^4^ cells/cm^2^. We then compared the effect of Dicer siRNA on luciferase activity derived from the two constructs using the Dual‐Glo Luciferase Assay System (Promega) according to the manufacturer's description.

The Dicer siRNA (s23755 by ThermoFisher Scientific) is not directed against ASIC2 3′UTR, however, it is expected to down‐regulate the accumulation of all miRNAs since Dicer is required for the biogenesis of all miRNAs. Therefore, if the 3′UTR of the gene is targeted by any miRNA, in the absence of Dicer, we would observe a higher luciferase activity.

Each assay was repeated eight times (two independent experiments with four biological replicates each) and the firefly luciferase activity was normalized for the Renilla luciferase activity for each assay. Given the limited number of observations and the impossibility to verify the normality assumption, we analyzed data using a non‐parametric Wilcoxon rank sum test. The test was one‐sided, based on the *a priori* hypothesis that miRNA binding to the target sequence is expected to decrease gene expression, and hence luciferase activity.

## 3 RESULTS

### 3.1 ASICs involvement in EAE

**Figure 1:**
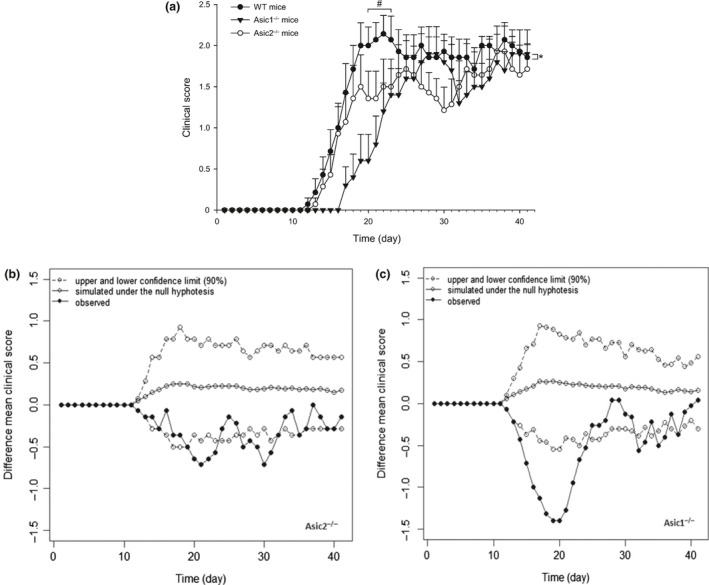
(a) Clinical score of mice after immunization with MOG_35‐55_ peptide. Values are means + *SEM* and data are cumulative of two independent experiments with similar results with a total of 14 wild‐type (WT), 10 Asic1^−/−^ and 14 Asic2^−/−^ mice. * *p* = 0.019 (strain factor); *p* < 0.001 (time factor); *p* < 0.001 (strain × time factor) vs. WT mice; # *p* = 0.013 for day 20; *p* = 0.004 for day 21; *p* = 0.013 for day 22; *p* = 0.032 for day 23 of WT mice vs. the respective values in Asic2^−/−^ mice (Two‐way ANOVA Repeated Measures + Bonferroni post hoc test). (b) Comparison between the observed difference between the mean clinical scores of Asic2^−/−^ and wild‐type (WT) and the pattern of difference simulated under the null hypothesis of no difference. (c) Comparison between the observed difference between the mean clinical scores of Asic1^−/−^ and wild‐type (WT) and the pattern of difference simulated under the null hypothesis of no difference. In (b) and (c) data points lying outside the lower confidence band can be interpreted as evidence of a departure from the null hypothesis of no difference

In order to study whether ASIC1 and ASIC2 have a disease‐modifying effect, thus suggesting an involvement in the pathogenesis of MS, we induced EAE on mice. MOG‐immunized WT mice showed a mean onset of the disease on day 16.7 ± 1, reached the peak of the clinical score on day 22 (cumulative score = 50.2 ± 4.3) with an incidence of 100%. Asic1^−/−^ mice showed a mean onset of the disease on day 23 + 1.9, reached the peak of the clinical score on day 28 (cumulative score = 35.5 + 4.8) with an incidence of 90%. Asic2^−/−^ mice showed a mean onset of the disease on day 20.8 + 2.2, reached the peak of the clinical score on day 37 (cumulative score = 40.1 + 6.1) with an incidence of 100%. Analysis of the clinical score in the three strains of mice showed that Asic1^−/−^ mice have a delayed disease onset and EAE peak, in line with the scientific literature (Friese et al., 2007), whereas Asic2^−/−^ mice showed a significant reduction of the clinical score only in days 20–23 after immunization (Figure 1a). To test if there was a significant difference between the profiles of the three strains of mice we computed the 90% empirical CIs. The latter were computed from the empirical distribution generated by permutation under the null hypothesis of no difference between the mean of clinical score of Asic1^−/−^ and WT and between the mean of clinical score of Asic2^−/−^ and WT. Data points lying outside the lower confidence band can be interpreted as evidence of a departure from the null hypothesis. We observed a series of consecutive data points lying below the lower band of 90% CI (Figure 1b and c). This pattern was much more pronounced for Asic1^−/−^ than for Asic2^−/−^. More specifically, in Asic1^−/−^ mice, the clinical score showed a statistically significant reduction from day 14 to day 24 since EAE induction; for Asic2^−/−^ the reduction is still statistically significant but from 20 to 23 days and the size of the reduction is a much less than for Asic1^−/−^ mice.

### 3.2 Histology and immunohistochemistry analysis of spinal cord of EAE mice

**Figure 2:**
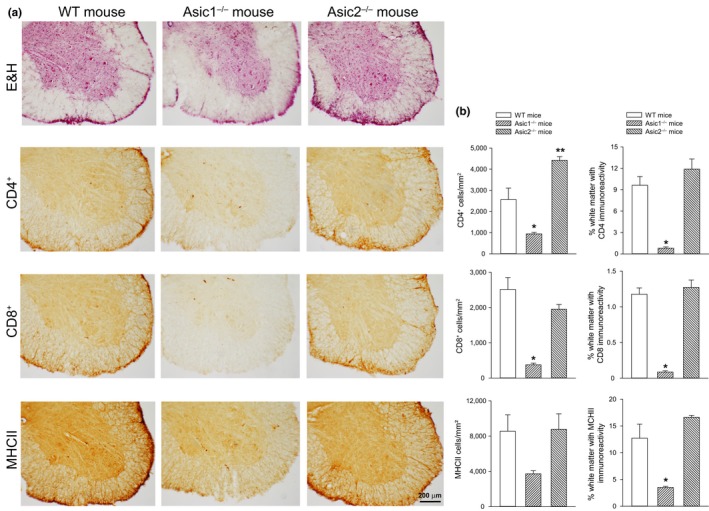
Histopathological analysis of spinal cord in EAE mice. (a) Representative spinal cord sections from wild‐type (WT), Asic1^−/−^ , and Asic2^−/−^ mice at 45 days after immunization. Sections of the spinal cord anterior horn were stained with hematoxylin and eosin (E&H) to assess inflammation and immunostained for MCHII, CD4^+^, CD8^+^ infiltrating cells. (b) Graphs show the quantification of the extent of inflammation and infiltrating MCHII, CD4^+^ and CD8^+^ cells in the anterior horn of the spinal cord. Values are means + *SEM* (*n* = 3 animals for each group). *p = 0.014 for CD4^+^ cells/mm^2^ vs. WT mice; ** *p* = 0.008 for CD4^+^ cells/mm^2^ vs. WT mice; *p* < 0.001 for % white matter with CD4^+^ immunoreactivity vs. WT mice; *p* < 0.001 for CD8^+^ cells/mm^2^ vs. WT mice; *p* < 0.001 for % white matter with CD8^+^ immunoreactivity vs. WT mice; *p* = 0.091 for MHCII cells/mm^2^ vs. WT mice; *p* < 0.002 for % white matter with MHCII immunoreactivity vs. WT mice (One‐way ANOVA + Fisher's LSD). [Colour figure can be viewed at wileyonlinelibrary.com]

Wild‐type mice immunized with MOG_35‐55_ showed adaptive immune cell infiltrates in the spinal cord, assessed at day 45 following immunization (Figure 2a). CD4^+^ and MHC class‐II^+^ mononuclear cells were largely predominant in the immune infiltrate, although CD8^+^ cytotoxic cells could also be detected. Quantification of the adaptive immune cell infiltrates in the spinal cord showed a significant reduction in Asic1^−/−^ mice which paralleled the reduced clinical score of EAE mice (Figure 2b). We observed an increase of CD4^+^ mononuclear cells in Asic2^−/−^ mice when the clinical score was similar to WT mice (Figure 2b).

### 3.3 ASICs involvement in pain

**Figure 3:**
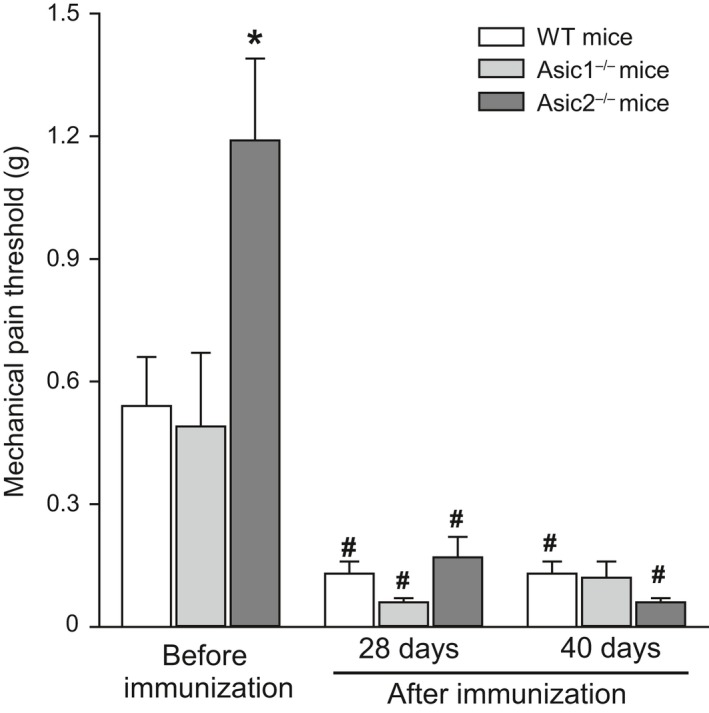
Mechanical allodynia assessment in mice. Mechanical pain thresholds were assessed in wild‐type (WT;* n* = 20), Asic1^−/−^ (*n* = 12) and Asic2^−/−^ (*n* = 21) mice under basal physiological conditions (before immunization), and in pathological conditions at 28 days and at 40 days after immunization with MOG_35‐55_ peptide. Values are means ± *SEM* and are cumulative of two independent experiments. * *p* < 0.001 vs. WT and Asic1^−/−^ mice under basal conditions (before immunization), and # *p* = 0.007 WT mice at 28 days vs. WT mice before immunization; *p* = 0.025 Asic1^−/−^ mice at 28 days vs. Asic1^−/−^ mice before immunization; *p* < 0.001 Asic2^−/−^ mice at 28 days vs. Asic2^−/−^ mice before immunization; *p* = 0.016 WT mice at 40 days vs. WT mice before immunization; *p* < 0.001 Asic2^−/−^ mice at 40 days vs. Asic2^−/−^ mice before immunization (One‐way ANOVA + Fisher's LSD)

To assess the involvement of ASIC1 and ASIC2 in pain and if EAE induction promotes any differential change, we measured mechanical pain thresholds in WT, Asic1^−/−^ and Asic2^−/−^ mice under basal physiological conditions and in pathological conditions at 28 and 40 days after immunization. It is noteworthy under physiological conditions, i.e., before immunization, in Asic2^−/−^, we observed a significant increased mechanical pain threshold as compared to Asic1^−/−^ and WT mice (Figure 3). After immunization, WT and Asic1^−/−^ mice showed a reduced mechanical pain threshold by about 4–5 fold, but, to our surprise, Asic2^−/−^ mice also showed a reduced mechanical pain threshold by about 6 fold, likely due to the partial paralysis of posterior legs that may have occluded the increased pain threshold observed before EAE in these mice.

### 3.4 ASIC2 mRNA expression is increased in MS brain

**Figure 4:**
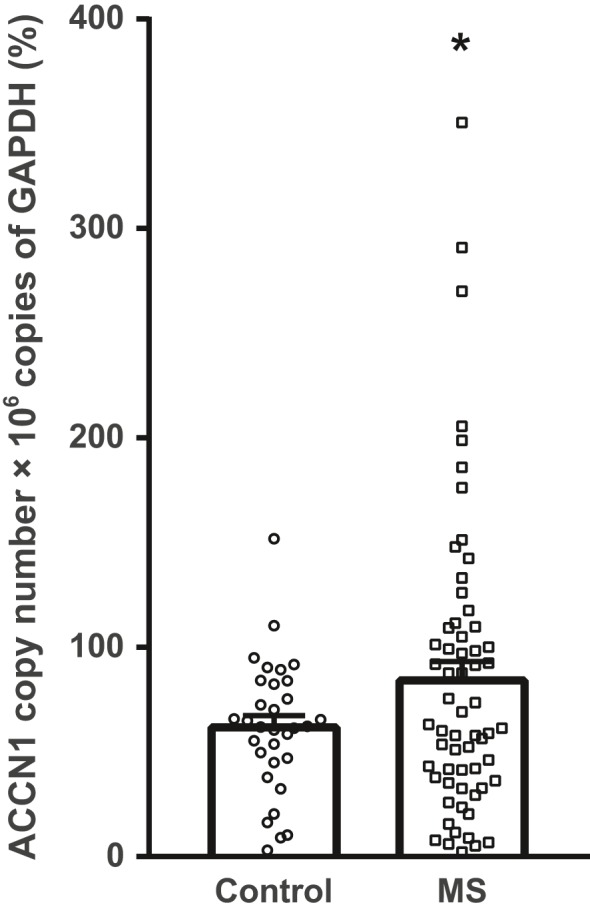
ASIC2 mRNA levels in the brain of 32 control cases and 62 multiple sclerosis (MS) cases. Single values are shown along with means ± *SEM* expressed as % to cumulate data coming from different quantitative PCR runs. * *p* = 0.0457 (Student')s *t* test)

ASIC2 expression was quantified in human brain tissues of 62 cases with secondary progressive MS and 32 controls (affected by other non‐neurological disease). Quantitative real‐time PCR analysis showed a significant increase of ASIC2 transcript in brain tissues of MS cases compared to control cases (Figure 4), suggesting a role of ASIC2 in the pathophysiology of MS, while we did not observe evidence of a significant effect of rs28936 genotype under the assumption of a recessive model, on ASIC2 expression level possibly due to the small sample size (*p* = 0.11, *β* = −22.14 [−49.53; 5.24]).

We also analyzed the association between rs28936 and MS in brain samples (*β* = 1.02 [−0.02; 2.21], *p* = 0.068). This borderline significance is probably due to the small sample size.

Rs28936 was analyzed, assuming a genetic recessive model (only AA genotype will increase disease risk), after testing for the departure from the additive model. *p* < 0.05 on 2‐sided test was considered as statistically significant. The means ± *SEM* of the expression data of the subject carried, respectively, the AA genotype and the GA/GG genotypes are 62.5 ± 5.19 and 79.2 ± 5.70.

### 3.5 ASIC2 3′UTR regulation is Dicer dependent

Next, we performed a luciferase report gene assay to investigate whether ASIC2 expression is under miRNAs regulation dependent on rs28936 genotype. All the results are presented in Table 1.

**Table 1:** Luciferase reporter gene assay results

AA construct, Dicer siRNA Median (IQR)	AA construct, scrambled siRNA Median (IQR)	*p*‐Value	GG construct, Dicer siRNA Median (IQR)	GG construct, scrambled siRNA Median (IQR)	*p*‐Value
11.05 (10.27–11.43)	6.07 (5.51–6.52)	3.51*10^−7^	16.71 (15.90–18.35)	14.29 (13.49–15.58)	7.42*10^−5^

IQR, Interquartile Range. Luciferase activity for each group (siRNA/SiRNA scramble) and for each allele (A/G) of rs28936 was compared by means of a one‐sided Wilcoxon rank‐sum test. Descriptive measures are median and interquartile range.

We found that the rs28936A construct showed a higher increase in luciferase activity than the rs28936G construct in the presence of Dicer siRNA (*p* = 3.51 × 10^−7^). The increase represents the difference between activity in the presence of the Dicer siRNA and scrambled siRNA.

This indicates that rs28936A ASIC2 mRNA is under stronger miRNA regulation than rs28936G, as luciferase activity is more increased when the global miRNA level is lower. However, the rs28936G is also under a milder control, with an increase of luciferase activity which is statistically different from zero (*p* = 7.42 × 10^−5^), suggesting that both rs28936 alleles are under the control of microRNAs.

## 4 DISCUSSION

The genetic association between rs28936 in the 3′UTR of ASIC2 and MS observed in Nuoro population (Bernardinelli et al., 2007), the evidence that ASIC1 is involved in the pathogenesis of EAE on animal model (Friese et al., 2007) and the altered expression of sodium channels reported in MS (Waxman, 2001) lead us to hypothesize that ASIC2, with which ASIC1 interacts forming heteromultimeric channels, could be an important contributor to neuropathological processes in MS.

Although ASIC2 subunits are widely expressed in brain and modulate ASIC1a current, their function is not yet clear. To investigate our hypothesis of an involvement of ASIC2 in MS, we carried out different experiments, both *in vivo* and *in vitro*. In the first *in vivo* experiment we analysed clinical scores in WT, Asic2^−/−^ and Asic1^−/−^ mice with induced EAE at different times from immunization. As expected, after induction of EAE, mice lacking Asic1 showed an attenuation of the clinical score over time compared to WT. These effects were also observed in Asic2^−/−^ mice but in a limited time window. Immunohistochemistry for MHCII, CD4^+^ and CD8^+^ on spinal cord of EAE mice showed a significant reduction of inflammatory infiltrates in ASIC1^−/−^ mice paralleling the clinical score. An increase of CD4^+^ mononuclear cells was observed in Asic2^−/−^ mice, whereas MHCII and CD8^+^ were not modified as compared to WT mice. We do not have any explanation for this effect. Overall, these results suggest that despite the fact that ASIC1 seems to play a major role in EAE severity, ASIC2 is also involved, probably as a consequence of the physical interaction existing between the two channels.

The pH changes in inflamed tissue led to hypothesize that local acidosis may be involved in pain and given the involvement of ASICs in pain modulation (Wemmie et al., 2013), we analyzed mechanical pain thresholds in WT, Asic2^−/−^ and Asic1^−/−^ mice under physiological conditions, i.e., before immunization, and at different time points after immunization. We observed an increased pain threshold in Asic2^−/−^ mice in physiological conditions, as compared to Asic1^−/−^ and WT. Our results are in line with the observation that Asic1^−/−^ mice do not show alteration of skin mechanoreceptor function in physiological conditions (Page et al., 2004) and are in contrast with data reported by others (Price et al., 2000; Roza et al., 2004; Staniland & McMahon, 2009). In this latter case the authors obtained data from an in vitro skin/nerve preparation (Price et al., 2000; Roza et al., 2004), which is a different condition than the more complex in vivo situation. The observations that triple knockout mice for Asic1a, Asic2 and Asic3, in physiological conditions, have an increased pain sensitivity (Kang et al., 2012), and the lack of increased pain threshold in Asic2^−/−^ mice (Staniland & McMahon, 2009), which contrasts with our results, may suggest complex interactions between these three cation channels and other proteins in modulating pain transmission. In fact, ASICs interact with multiple scaffolding proteins, as PSD‐95 (Zha et al., 2009) and ion channels linked to mechanosensation, as Big Potassium channels (Petroff et al., 2008). We observed a statistically significant reduction of mechanical pain thresholds in all strains of mice after EAE. However, other models of acute and chronic inflammatory pain and neuropathic pain may help to understand the role of ASICs in pain processing and pain matrix.

Our investigation on the role of ASIC2 and rs28936, with regard to an allelic‐specific expression, led us to analyze the transcript of this gene in autopsy brains of MS cases and controls. The quantitative analysis showed an increased expression of ASIC2 in MS brains, but we found only a weak statistical evidence of rs28936 allele‐specific effect (*p* = 0.11), possibly due to a lack of power.

Given that ASIC1a activation triggers the intracellular accumulation of Na^+^ and Ca^2+^ involved in neurodegenerative and inflammatory processes typical of MS, an overexpression of ASIC2 in MS brain could facilitate the heteromultimerization of ASIC2 subunits with ASIC1a; thus, resulting in an increase of channel expression at the cell surface and consequently of acid‐evoked current. When ASIC2a heteromultimerizes with other subunits, it reduces pH sensitivity. Furthermore, ASIC2 subunits facilitate subcellular localization of ASIC channels (Harding, Kusama, Hattori, Gautam, & Benson, 2014) through the interaction between ASIC2 and the neuronal scaffolding protein PSD‐95, thus increasing ASIC1a localization in dendritic spines (Zha et al., 2009).

Luciferase report assay on MCF7 cells showed a higher increase in luciferase activity for rs28936A than rs28926G in the presence of Dicer siRNA compared to scrambled siRNA treatment, highlighting that rs28936A is under a stronger miRNA regulation.

The impact of miRNAs regulation in MS has started to emerge in the last few years, and a few miRNAs have been found differentially expressed in MS lesions. An interesting example is represented by hsa‐miR‐27a, whose expression was found upregulated in active MS lesions (Junker et al., 2009). Hsa‐miR‐27a is induced in response to hypoxia (Camps et al., 2014), which is a well‐known feature of inflammation leading to acidosis, which in turn opens ASICs channels. We hence may hypothesized that the induction of this miRNA in presence of acidosis could decrease the expression of ASIC2 and it may results in a lower response to acidosis and inflammation, thus exerting a possible protective effect.

In conclusion, the strength of our study stands in the use of complementary approaches (*in vivo, in vitro,* and human brain samples) to characterize ASIC2 contribution in MS pathophysiology and the integration of different evidences from experimental data on the role of ASIC2 in MS, which was previously limited to ASIC1. The significant reduction of the clinical score in Asic2^−/−^ mice in a limited time window, and, more importantly, the increased expression of ASIC2 in human brain MS samples, despite some limitations, suggests a possible role of ASIC2 in the pathophysiology of MS even if additional studies are required to firmly establish it.

## CONFLICT OF INTEREST

The authors declared no conflicts of interest.

## DATA ACCESSIBILITY

All data including animal scores, microscopy images, cell counts and mRNA levels are maintained at Neuromed Institute. All data can be accessed by contacting the corresponding Author at luisa.bernardinelli@unipv.it.

## AUTHOR CONTRIBUTIONS

SN, CB, TI, MC, DG and TD performed experiments. SN, CB, TI, MC, TF, RP and GM analyzed data. LB, GB, CB, TD, AT, PB were involved in study concept and design. TF, LB, GB, CB, AT and PB wrote the manuscript. All the authors were involved in the interpretation of the results, read, critically revised and approved the final manuscript before submission.

## References

[ejn14302-bib-0001] Arun, T. , Tomassini, V. , Sbardella, E. , de Ruiter, M. B. , Matthews, L. , Leite, M. I. , … Palace, J. (2013). Targeting ASIC1 in primary progressive multiple sclerosis: Evidence of neuroprotection with amiloride. Brain, 136, 106–115. 10.1093/brain/aws325 23365093

[ejn14302-bib-0002] Bartoi, T. , Augustinowski, K. , Polleichtner, G. , Gründer, S. , & Ulbrich, M. H. (2014). Acid‐sensing ion channel (ASIC) 1a/2a heteromers have a flexible 2:1/1:2 stoichiometry. Proceedings of the National Academy of Sciences of the United States of America, 111, 8281–8286. 10.1073/pnas.1324060111 24847067 PMC4050600

[ejn14302-bib-0003] Beecham, A. H. , Patsopoulos, N. A. , Xifara, D. K. , Davis, M. F. , Kemppinen, A. , Cotsapas, C. , … McCauley, J. L. (2013). Analysis of immune‐related loci identifies 48 new susceptibility variants for multiple sclerosis. Nature Genetics, 45, 1353–1360. 10.1038/ng.2770 24076602 PMC3832895

[ejn14302-bib-0004] Bernardinelli, L. , Murgia, S. B. , Bitti, P. P. , Foco, L. , Ferrai, R. , Musu, L. , … Berzuini, C. (2007). Association between the ACCN1 gene and multiple sclerosis in central east Sardinia. PLoS ONE, 2(5), e480 10.1371/journal.pone.0000480 17534430 PMC1868958

[ejn14302-bib-0005] Boscardin, E. , Alijevic, O. , Hummler, E. , Frateschi, S. , & Kellenberger, S. (2016). The function and regulation of acid‐sensing ion channels (ASICs) and the epithelial Na(+) channel (ENaC): IUPHAR Review 19. British Journal of Pharmacology, 173, 2671–2701. 10.1111/bph.13533 27278329 PMC4995293

[ejn14302-bib-0006] van Buuren, S. , & Groothuis‐Oudshoorn, K. (2011). mice: Multivariate imputation by chained equations in R. Journal of Statistical Software, 45, 1233–67.

[ejn14302-bib-0007] Camps, C. , Saini, H. K. , Mole, D. R. , Choudhry, H. , Reczko, M. , Guerra‐Assunção, J. A. , … Ragoussis, J. (2014). Integrated analysis of microRNA and mRNA expression and association with HIF binding reveals the complexity of microRNA expression regulation under hypoxia. Molecular Cancer, 13, 28 10.1186/1476-4598-13-28 24517586 PMC3928101

[ejn14302-bib-0008] Chaplan, S. R. , Bach, F. W. , Pogrel, J. W. , Chung, J. M. , & Yaksh, T. L. (1994). Quantitative assessment of tactile allodynia in the rat paw. Journal of Neuroscience Methods, 53, 55–63. 10.1016/0165-0270(94)90144-9 7990513

[ejn14302-bib-0009] Consortium, I. M. S. G. , Consortium, W. T. C. C. , Sawce, S. , Hellenthal, G. , Pirinen, M. , Spencer, C. C. , … Compston, A. (2011). Genetic risk and a primary role for cell‐mediated immune mechanisms in multiple sclerosis. Nature, 476, 214–219. 10.1038/nature10251 21833088 PMC3182531

[ejn14302-bib-0010] Dendrou, C. A. , Fugger, L. , & Friese, M. A. (2015). Immunopathology of multiple sclerosis. Nature Reviews Immunology, 15, 545–558. 10.1038/nri3871 26250739

[ejn14302-bib-0011] Du, J. , Reznikov, L. R. , Price, M. P. , Zha, X. , Lu, Y. , Moninger, T. O. , … Welsh, M. J. (2014). Protons are a neurotransmitter that regulates synaptic plasticity in the lateral amygdala. Proceedings of the National Academy of Sciences of the United States of America, 111, 8961–8966. 10.1073/pnas.1407018111 24889629 PMC4066526

[ejn14302-bib-0012] Fallarino, F. , Volpi, C. , Fazio, F. , Notartomaso, S. , Vacca, C. , Busceti, C. , … Di Marco, R. (2010). Metabotropic glutamate receptor‐4 modulates adaptive immunity and restrains neuroinflammation. Nature Medicine, 16, 897–902. 10.1038/nm.2183 20657581

[ejn14302-bib-0013] Friese, M. A. , Craner, M. J. , Etzensperger, R. , Vergo, S. , Wemmie, J. A. , Welsh, M. J. , … Fugger, L. (2007). Acid‐sensing ion channel‐1 contributes to axonal degeneration in autoimmune inflammation of the central nervous system. Nature Medicine, 13, 1483–1489. 10.1038/nm1668 17994101

[ejn14302-bib-0014] Harding, A. M. S. , Kusama, N. , Hattori, T. , Gautam, M. , & Benson, C. J. (2014). ASIC2 subunits facilitate expression at the cell surface and confer regulation by PSD‐95. PLoS ONE, 9, e93797 10.1371/journal.pone.0093797 24699665 PMC3974781

[ejn14302-bib-0015] Hoppenbrouwers, I. A. , & Hintzen, R. Q. (2011). Genetics of multiple sclerosis. Biochimica et Biophysica Acta, 1812, 194–201. 10.1016/j.bbadis.2010.09.017 20933079

[ejn14302-bib-0016] Jagodic, M. , Becanovic, K. , Sheng, J. R. , Wu, X. , Bäckdahl, L. , Lorentzen, J. C. , … Olsson, T. (2004). An advanced intercross line resolves Eae18 into two narrow quantitative trait loci syntenic to multiple sclerosis candidate loci. Journal of Immunology, 173, 1366–1373. 10.4049/jimmunol.173.2.1366 15240732

[ejn14302-bib-0017] Junker, A. , Krumbholz, M. , Eisele, S. , Mohan, H. , Augstein, F. , Bittner, R. , … Meinl, E. (2009). MicroRNA profiling of multiple sclerosis lesions identifies modulators of the regulatory protein CD47. Brain, 132, 3342–3352. 10.1093/brain/awp300 19952055

[ejn14302-bib-0018] Kang, S. , Jang, J. H. , Price, M. P. , Gautam, M. , Benson, C. J. , Gong, H. , … Brennan, T. J. (2012). Simultaneous disruption of mouse ASIC1a, ASIC2 and ASIC3 genes enhances cutaneous mechanosensitivity. PLoS ONE, 7, e35225 10.1371/journal.pone.0035225 22506072 PMC3323639

[ejn14302-bib-0019] Kreple, C. J. , Lu, Y. , Taugher, R. J. , Schwager‐Gutman, A. L. , Du, J. , Stump, M. , … Wemmie, J. A. (2014). Acid‐sensing ion channels contribute to synaptic transmission and inhibit cocaine‐evoked plasticity. Nature Neuroscience, 17, 1083–1091. 10.1038/nn.3750 24952644 PMC4115047

[ejn14302-bib-0020] Kweon, H.‐J. , & Suh, B.‐C. (2013). Acid‐sensing ion channels (ASICs): Therapeutic targets for neurological diseases and their regulation. BMB Reports, 46, 295–304. 10.5483/BMBRep.2013.46.6.121 23790972 PMC4133903

[ejn14302-bib-0021] Lill, C. M. , Luessi, F. , Alcina, A. , Sokolova, E. A. , Ugidos, N. , de la Hera, B. , … Bertram, L. (2015). Genome‐wide significant association with seven novel multiple sclerosis risk loci. Journal of Medical Genetics, 52, 848–855. 10.1136/jmedgenet-2015-103442 26475045

[ejn14302-bib-0022] Moutsianas, L. , Jostins, L. , Beecham, A. H. , Dilthey, A. T. , Xifara, D. K. , Ban, M. , … McVean, G. (2015). Class II HLA interactions modulate genetic risk for multiple sclerosis. Nature Genetics, 47, 1107–1113. 10.1038/ng.3395 26343388 PMC4874245

[ejn14302-bib-0023] Ortega‐Ramírez, A. , Vega, R. , & Soto, E. (2017). Acid‐sensing ion channels as potential therapeutic targets in neurodegeneration and neuroinflammation. Mediators of Inflammation, 2017, 3728096. 29056828 10.1155/2017/3728096 PMC5625748

[ejn14302-bib-0024] Page, A. J. , Brierley, S. M. , Martin, C. M. , Martinez‐Salgado, C. , Wemmie, J. A. , Brennan, T. J. , … Blackshaw, L. A. (2004). The ion channel ASIC1 contributes to visceral but not cutaneous mechanoreceptor function. Gastroenterology, 127, 1739–1747. 10.1053/j.gastro.2004.08.061 15578512

[ejn14302-bib-0025] Patsopoulos, N. A. , Esposito, F. , Reischl, J. , Lehr, S. , Bauer, D. , Heubach, J. , … de Bakker, P. I. W. (2011). Genome‐wide meta‐analysis identifies novel multiple sclerosis susceptibility loci. Annals of Neurology, 70, 897–912. 10.1002/ana.22609 22190364 PMC3247076

[ejn14302-bib-0026] Petroff, E. Y. , Price, M. P. , Snitsarev, V. , Gong, H. , Korovkina, V. , Abboud, F. M. , & Welsh, M. J. (2008). Acid‐sensing ion channels interact with and inhibit BK K+ channels. Proceedings of the National Academy of Sciences of the United States of America, 105, 3140–3144. 10.1073/pnas.0712280105 18287010 PMC2268598

[ejn14302-bib-0027] Price, M. P. , Gong, H. , Parsons, M. G. , Kundert, J. R. , Reznikov, L. R. , Bernardinelli, L. , … Welsh, M. J. (2014). Localization and behaviors in null mice suggest that ASIC1 and ASIC2 modulate responses to aversive stimuli. Genes, Brain, and Behavior, 13, 179–194. 10.1111/gbb.12108 PMC3998777 24256442

[ejn14302-bib-0028] Price, M. P. , Lewin, G. R. , McIlwrath, S. L. , Cheng, C. , Xie, J. , Heppenstall, P. A. , … Welsh, M. J. (2000). The mammalian sodium channel BNC1 is required for normal touch sensation. Nature, 407, 1007–1011. 10.1038/35039512 11069180

[ejn14302-bib-0029] Radu, B. M. , Dumitrescu, D. I. , Marin, A. , Banciu, D. D. , Iancu, A. D. , Selescu, T. , & Radu, M. (2014). Advanced type 1 diabetes is associated with ASIC alterations in mouse lower thoracic dorsal root ganglia neurons. Cell Biochemistry and Biophysics, 68, 9–23. 10.1007/s12013-013-9678-5 23723009

[ejn14302-bib-0030] Rajamäki, K. , Nordström, T. , Nurmi, K. , Åkerman, K. E. O. , Kovanen, P. T. , Öörni, K. , & Eklund, K. K. (2013). Extracellular acidosis is a novel danger signal alerting innate immunity via the NLRP3 inflammasome. Journal of Biological Chemistry, 288, 13410–13419. 10.1074/jbc.M112.426254 23530046 PMC3650379

[ejn14302-bib-0031] Roza, C. , Puel, J.‐L. , Kress, M. , Baron, A. , Diochot, S. , Lazdunski, M. , & Waldmann, R. (2004). Knockout of the ASIC2 channel in mice does not impair cutaneous mechanosensation, visceral mechanonociception and hearing. Journal of Physiology, 558, 659–669. 10.1113/jphysiol.2004.066001 15169849 PMC1664970

[ejn14302-bib-0032] Sherwood, T. W. , Lee, K. G. , Gormley, M. G. , & Askwith, C. C. (2011). Heteromeric acid‐sensing ion channels (ASICs) composed of ASIC2b and ASIC1a display novel channel properties and contribute to acidosis‐induced neuronal death. Journal of Neuroscience, 31, 9723–9734. 10.1523/JNEUROSCI.1665-11.2011 21715637 PMC3160670

[ejn14302-bib-0033] Staniland, A. A. , & McMahon, S. B. (2009). Mice lacking acid‐sensing ion channels (ASIC) 1 or 2, but not ASIC3, show increased pain behaviour in the formalin test. European Journal of Pain, 13, 554–563. 10.1016/j.ejpain.2008.07.001 18801682

[ejn14302-bib-0034] Tong, J. , Wu, W.‐N. , Kong, X. , Wu, P.‐F. , Tian, L. , Du, W. , … Gong, F. (2011). Acid‐sensing ion channels contribute to the effect of acidosis on the function of dendritic cells. Journal of Immunology, 186, 3686–3692. 10.4049/jimmunol.1001346 21321108

[ejn14302-bib-0035] Vergo, S. , Craner, M. J. , Etzensperger, R. , Attfield, K. , Friese, M. A. , Newcombe, J. , … Fugger, L. (2011). Acid‐sensing ion channel 1 is involved in both axonal injury and demyelination in multiple sclerosis and its animal model. Brain, 134, 571–584. 10.1093/brain/awq337 21233144

[ejn14302-bib-0036] Waldmann, R. , Champigny, G. , Voilley, N. , Lauritzen, I. , & Lazdunski, M. (1996). The mammalian degenerin MDEG, an amiloride‐sensitive cation channel activated by mutations causing neurodegeneration in Caenorhabditis elegans. Journal of Biological Chemistry, 271, 10433–10436. 10.1074/jbc.271.18.10433 8631835

[ejn14302-bib-0037] Waxman, S. G. (2001). Transcriptional channelopathies: An emerging class of disorders. Nature Reviews Neuroscience, 2, 652–659. 10.1038/35090026 11533733

[ejn14302-bib-0038] Wemmie, J. A. , Taugher, R. J. , & Kreple, C. J. (2013). Acid‐sensing ion channels in pain and disease. Nature Reviews Neuroscience, 14, 461–471. 10.1038/nrn3529 23783197 PMC4307015

[ejn14302-bib-0039] Wu, J. , Leng, T. , Jing, L. , Jiang, N. , Chen, D. , Hu, Y. , … Zha, X. (2016). Two di‐leucine motifs regulate trafficking and function of mouse ASIC2a. Molecular Brain, 9, 9 10.1186/s13041-016-0190-x 26819004 PMC4729175

[ejn14302-bib-0040] Wu, J. , Xu, Y. , Jiang, Y.‐Q. , Xu, J. , Hu, Y. , & Zha, X. (2016). ASIC subunit ratio and differential surface trafficking in the brain. Molecular Brain, 9, 4 10.1186/s13041-016-0185-7 26746198 PMC4706662

[ejn14302-bib-0041] Zha, X. , Costa, V. , Harding, A. M. S. , Reznikov, L. , Benson, C. J. , & Welsh, M. J. (2009). ASIC2 subunits target acid‐sensing ion channels to the synapse via an association with PSD‐95. Journal of Neuroscience, 29, 8438–8446. 10.1523/JNEUROSCI.1284-09.2009 19571134 PMC2734339

[ejn14302-bib-0042] Zhou, R.‐P. , Wu, X.‐S. , Wang, Z.‐S. , Xie, Y.‐Y. , Ge, J.‐F. , & Chen, F.‐H. (2016). Novel insights into acid‐sensing ion channels: Implications for degenerative diseases. Aging and Disease, 7, 491–501. 10.14336/AD.2015.1213 27493834 PMC4963192

