# Supplementary material for: Acid sensing ion channel 2: A new potential player in the pathophysiology of multiple sclerosis
Source: Eur J Neurosci. 2019 Feb 19;49(10):1233–43. doi: 10.1111/ejn.14302 (PMC6618268; doi:10.1111/ejn.14302)
Supplement: Supplementary file 1 [file EJN-49-1233-s001.pdf]

EJN European Journal of Neuroscience FENS Federation of European Neuroscience Societies

Acid Sensing Ion Channel 2: a new potential player in the pathophysiology of multiple sclerosis

Bernardinelli, Luisa; Fazia, Teresa; Pastorino, Roberta; Notartomaso, Serena; Busceti, Carla; Imbriglio, Tiziana; Cannella, Milena; Gentilini, Davide; Morani, Gabriele; Ticca, Anna; Bitti, Pier Paolo; Berzuini, Carlo; Dalmay, Tamas; Battaglia, Giuseppe

Review timeline:

Submission date: 17-Jul-18
Editorial Decision: Major Revision
Revision Received: 15-Oct-18
Editorial Decision: Minor Revision
Revision Received: 12-Nov-18
Accepted: 23-Nov-18

Editor: Yoland Smith
Reviewer 1: Sabatino Maione
Reviewer 2: Stephan Kellenberger
Reviewer 3: Enrique Soto

1st Editorial Decision 16-Aug-18

Dear Prof. Bernardinelli,

Your manuscript has been reviewed by three external reviewers as well as by the Section Editor, Dr. Yoland Smith, and ourselves. The reviews collectively indicate that your experiments generated new and important information. However, there are several issues that need to be clarified/resolved before we can consider your manuscript further for publication in EJN.

As you can see below, the three reviewers found significant merit to your study and consider that your findings are relevant to the field. However, two of them raised a few important points you need to pay attention to in the revised manuscript. The following issues are particularly relevant. 1) Please, clarify the presentation of data in figure 1 to address the concerns raised by one of the reviewers. 2) The rationale to look at ASIC2 instead of ASIC1 mRNA must be strengthened. 3) Describe in more detail the data collection approach for the behavioral experiments, paying a particular attention to the blinding of the observers to the animal genotype. We concur with the reviewer that the investigators must be blinded to the animal genotype. If this was not the case, it must be mentioned and caveats raised. 4) Revise discussion of EAE-induced lowering of the threshold for mechanical allodynia according to your findings and relate those to the previous literature.

We also note the following points that need to be addressed in the revised version.
- Provide a list of abbreviations.
- In accordance with EJN policy, please replace bar charts with more informative scatter plots, bar charts or similar.
- Indicate how the mice killed.
- How was 'percentage of white matter infiltrated' measured?
- Provide details of secondary antibodies.
- The English needs some improvement e.g. '..we induced EAE IN mice' Please carefully proof-read.

EJN European Journal of Neuroscience FENS Federation of European Neuroscience Societies

- Include statistical data in the text as well as the legends and enure it adheres to EJN guidelines, notably report precise P values.

When revising the manuscript, please embolden or underline major changes to the text so they are easily identifiable and please don't leave 'track change' formatting marks in your paper. Please ensure that you provide a text and a figure file for the Graphical Abstract (as detailed in the instructions below). When carrying out your revisions please refer to the checklist below and visit the EJN author guidelines at www.ejneuroscience.org

When finalized, please upload your complete revised manuscript onto the website, as a Word file (.doc, or .docx). Please also ensure that a complete set of tables and figures is included as separate files, even if these have not changed from the originals. At this stage it is necessary to provide high resolution figures. Please see important instructions below.

Please go into https://mc.manuscriptcentral.com/ejn - Author Centre - manuscripts with decisions where you will find a 'create a revision' link under 'actions'. We ask that you please indicate the way in which you have responded to the points raised by the Editors and Reviewers in a letter. Please upload this response letter as a separate Word (.doc or PDF) file using the file designation "Authors' Response to Reviewers" when uploading your manuscript files. Please DO NOT submit your revised manuscript as a new one. Also, please note that only the Author who submitted the original version of the manuscript should submit a revised version.

If you are able to respond fully to the points raised, we would be pleased to receive a revision of your paper within 12 weeks.

Thank you for submitting your work to EJN.

Best wishes,

Paul Bolam & John Foxe
co-Editors in Chief, EJN

Reviews:

Reviewer: 1

Comments to the Author
In this paper the authors examined the role of ASIC2 in the mouse model of experimental autoimmune encephalomyelitis (EAE) and autoptic human brain samples of controls and MS subjects. By using ASIC1 and ASIC2 knockout mice, they confirmed the role of ASIC1 in reducing the clinical score in EAE, already known in the scientific literature, but they also found that ASIC2 also plays a partial role in EAE. They also assessed pain thresholds in these knockout mice and showed that, in physiological conditions, ASIC2 is involved in pain processing. Moreover, they showed that ASIC2 mRNA levels are increased in human brain samples. Finally, by using an in vitro luciferase reporter gene assay, they assessed whether ASIC2 expression is under miRNA regulation.

EJN European Journal of Neuroscience FENS Federation of European Neuroscience Societies

Experimental procedures are solid and well carried out. The results obtained by combining in vitro and in vivo experimental paradigms are very well shown with clear statistical analysis, are very interesting, the conclusions drawn are appropriate and increase the knowledge of the role of ASICs in MS.

Minor points:
1. Figure 2 can be combined with Figure 1 as they show results on ASIC2 knockout mice in EAE.

A Figure showing the sequence alignment of siRNA and region on UTR would help to better appreciate the predicted targeting.
On page 5, line 13, Jensen et al., is repeated twice and one can be deleted.
On page 7, line 17, I guess it is “mice” instead of “rats”

Reviewer: 2

Comments to the Author
This study investigates the involvement of acid-sensing ion channels (ASICs), specifically ASIC1 and ASIC2, in some aspects of multiple sclerosis (MS) and experimental autoimmune encephalomyelitis (EAE). The authors show that the onset of the clinical phenotype of EAE is delayed in ASIC1(-/-) mice, and that the clinical score is slightly smaller in ASIC2(-/-) mice as compared to WT during the time window of day20-23. At day 45, the infiltration by MCHII-, CD4+- and CD8+-cells measured from spinal cord slices was significantly lower in ASIC1(-/-) but not ASIC2(-/-) when compared to WT. Before EAE induction, ASIC2(-/-) mice showed a higher threshold for mechanical allodynia than WT and ASIC1(-/-) mice. EAE induction led to significant lowering of this threshold in all genotypes, and there was no significant difference between genotypes in this regard. Analysis of ASIC2a mRNA expression from post mortem brain tissue of MS patients and controls showed a higher expression of ASIC2a mRNA in MS patients. The authors also mention their analysis of the involvement of variants in the ASIC2 UTR region on ASIC2 expression that was however not conclusive, and they provide in a cellular assay indirect evidence for a regulation of ASIC2 by miRNAs. This study shows different pieces of information concerning the relationship between ASICs and EAE in mice, and ASIC2 and MS in humans, that are potentially interesting, but are somewhat isolated from each other, are not sufficiently detailed and don’t make a logical, coherent story together. The work appears technically sound except for one aspect that needs to be clarified (point 2), and it adheres to the scope of this journal.

Major points
1. The authors try to focus in this study on ASIC2, since they have previously shown an association between a variant in the ASIC2 UTR and MS, and because there has been more research on the connection between MS/EAE and ASIC1, mostly by the laboratory of Lars Fugger. However, the results of the present study also point more strongly to ASIC1 (confirmation of delayed onset of EAE, infiltration in spinal cord). Based on these findings, it is not logical that the authors analyze the mRNA expression of ASIC2, but not ASIC1, in brain tissue of MS patients and controls. They were motivated to go in this direction by their previous finding of the association between MS and ASIC2, as mentioned above. It seems that in the present manuscript, on a different population than in the previously published study, this association could not be confirmed, however, the analysis is not even shown. The logics of the study asks for providing also the ASIC1 mRNA expression data, and to show the analysis of the association of MS with the different ASIC2a UTR variants. The authors need to find a way of presenting a coherent, logical manuscript. This may also be possible by further developing the analysis of the regulation by mi-RNA.
2. For the two types of behavioral experiment, the clinical signs of EAE, and the mechanical allodynia, it is

critical that the researchers carrying out the experiment are blinded to the genotype of the animal. There is however no mention of this in the Methods. The authors need to state in the methods whether the experimenters were blinded to the mouse genotype or not.
3. The mechanical allodynia experiments showed a higher threshold for ASIC2(-/-) mice than others under control conditions, and a lowering of the threshold by EAE in all three genotypes. There is no evidence provided for a statistically different effect of EAE on mechanical allodynia between genotypes. In the discussion (upper part of p.14) this observation is discussed as if the EAE-induced lowering of the threshold for mechanical allodynia was different in ASIC2(-/-) mice as compared to WT. This is however not shown. In the discussion of the increased threshold in ASIC2(-/-) mice under control conditions (discussion on lower part of p13), the authors should also mention studies that have carried out the same type of experiments (von Frey filaments on hind paw) and did not observe any difference between ASIC2(-/-) and WT, as for example Staniland et al., Eur J Pain (2009), and they should discuss possible reasons for this difference.

Specific points
1. The final statement of the abstract is too strong; the data provide only a weak support for a role of ASIC2 in MS.
2. P3, center of page, "ASIC2….involved in neurodegeneration in C. elegans…". This statement is wrong, degenerins, not ASIC2a are involved in neurodegeneration in C. elegans.
3. P5, lower part of first paragraph, "…showed that 4-aminopyridine, …inhibits heterotrimeric ASIC channels". This information should not be pushed too much as an evidence for an involvement of ASICs in MS, since clinical concentrations of 4-aminopyridine reached in MS therapy are below 1 microM, and the IC50 for ASIC inhibition by 4-aminopyridine is close to 1 mM.
4. P12, top paragraph, analysis of rs28936 genotype link to MS; this analysis should be shown.
5. P15, upper half, "The induction of has-miR-27a…" This statement and much of this paragraph needs to be declared more clearly as a hypothesis/speculation.
6. P15, bottom, "The increased pain thresholds in mice……suggests a possible role of ASIC2a in the pathophysiology of MS…". It is not correct to use results of the mechanical allodynia as an argument for an involvement of ASIC2 in EAE or MS. The study in its present form does not show evidence for a difference between ASIC2(-/-) mice and WT in EAE-induced pain.
7. Table 1, title of the 4th column from left, "AA construct, Dicer.." should probably be "GG construct, Dicer.."
8. The introduction and discussion are very long and should be shortened.

Reviewer: 3

Comments to the Author

(ASICs) Authors investigated the potential involvement of Acid Sensing Ion Channel-2 (ASIC2) in MS inflammatory process induced by autoimmune encephalomyelitis (EAE) in wild-type (WT), knockout Asic1-/- and Asic2-/- mice. They observed a significant reduction of clinical score in Asic1-/- mice and a significant reduction of the clinical score in Asic2-/- mice in a limited time window (20-23 days after immunization). Analysis of mechanical allodynia, showed a significant higher pain threshold in Asic2-/- and Asic1-/-. Findings suggest a role of ASIC2 in the pathophysiology of MS

EJN European Journal of Neuroscience FENS Federation of European Neuroscience Societies

Major
The results shown in figure 1 can be somewhat confusing, after reading it several times you understand what they mean, what really confuses me is that the authors refer the maximum clinical score peak for Asic1- / and Asic2- / - on day 27 and 25 respectively, but checking those days in the graph do not seem to coincide with the maximum values, for example in the case of Asic2 - / - I notice the maximum value in the clinical assessment on day 36.

Minor
Page 2 row 4. Specify that "rs28936" refers a Single Nucleotide Polymorphisms.
Page 4 row 2. They talk about the role of ASIC2a but they never mention the characteristics and functions of the ASIC2b subunit.
Page 5 row 7. Why do they use lowercase letters in "Asic1"?
Page 11 row 12. The significant increase of CD4+ in the Asic2 - / - mice is not mentioned
Page 11 row 19. Why do they use lowercase letters in "Asic1"?
Page 13 row 15. They do not discuss the increase of CD4+ that is observed in the infiltrate present in Asic2 - / - mice.
Page 15 row 24. Specify "The increased pain thresholds in Asic2-/- mice"
Figure 2 is not very readable, I recommend increasing its size.

Authors' Response 15-Oct-18

Dear Editor, Dear Reviewers,

Please, find enclosed the revised version of manuscript number EJN-2018-07-25771, which we prepared in the light of the Reviewers' comments.

We are very grateful to the reviewers for their helpful feedback on this manuscript.

Prof. Luisa Bernardinelli

In accordance with the EJN policy:

- List of abbreviation is provided at the end of the manuscript;

- Graph of Figure 5 has been changed;

- We now report how mice were killed in the paragraph "*Histology and immunohistochemistry analysis of spinal cord of EAE mice*";
- We now report how percentage of infiltrated white matter was measured at the end of the paragraph "*Histology and immunohistochemistry analysis of spinal cord of EAE mice*";
- Details on secondary antibodies are reported in the paragraph "*Histology and immunohistochemistry analysis of spinal cord of EAE mice*";
- The English has been improved;
- Exact values of P values are now reported and statistical data were included in the text as well as the legends;
- We have uploaded new figures.

Below we discuss the Reviewers' comment in a point-by-point fashion.

<u>Reviewer #1:</u>

Figure 2 can be combined with Figure 1 as they show results on ASIC2 knockout mice in EAE.
We produced a new figure called Figure 1 comprising the two original figures.

A Figure showing the sequence alignment of siRNA and region on UTR would help to better appreciate the predicted targeting.
ASIC2 3'UTR was cloned downstream of the firefly luciferase gene, however there is not a direct linkage between SiRNA and the region on ASIC2 3'UTR. The siRNA is directed against the gene Dicer. Dicer silencing is expected to down-regulate the accumulation of all miRNAs since Dicer is required for the biogenesis of all miRNAs. Therefore, if the 3'UTR of the gene is targeted by any miRNA, in the absence of Dicer we would observe a higher luciferase activity. For this reason we cannot produce a figure showing a sequence

consensus of siRNA and region on UTR. The materials and methods section has been rephrased in order to clarify this issue.

On page 5, line 13, Jensen et al., is repeated twice and one can be deleted.
We decided to eliminate this sentence, also in an effort to simply the introduction.

On page 7, line 17, I guess it is “mice” instead of “rats”.
Done.

<u>Reviewer #2:</u>

Major points:

1. The authors try to focus in this study on ASIC2, since they have previously shown an association between a variant in the ASIC2 UTR and MS, and because there has been more research on the connection between MS/EAE and ASIC1, mostly by the laboratory of Lars Fugger. However, the results of the present study also point more strongly to ASIC1 (confirmation of delayed onset of EAE, infiltration in spinal cord). Based on these findings, it is not logical that the authors analyze the mRNA expression of ASIC2, but not ASIC1, in brain tissue of MS patients and controls. They were motivated to go in this direction by their previous finding of the association between MS and ASIC2, as mentioned above. It seems that in the present manuscript, on a different population than in the previously published study, this association could not be confirmed, however, the analysis is not even shown. The logics of the study asks for providing also the ASIC1 mRNA expression data, and to show the analysis of the association of MS with the different ASIC2a UTR variants. The authors need to find a way of presenting a coherent, logical manuscript. This may also be possible by further developing the analysis of the regulation by mi-RNA

We gratefully thank the reviewer for this comment that allow us to better clarify the logic underlying our study.
In this study, we started from the evidence of a statistically significant association between the SNP rs28936 and MS in Sardinian families, a result we obtained in our previous study in which we analyzed also other ASIC2 variants. Rs28936, turned to be the most statistically significant one, is localized on 3’UTR of ASIC2 and this has motivated our investigation to be focused on this gene, being 3’UTR a region involved in the regulation of gene expression also possibly driven by miRNA. For this reason, one of our aims was to understand if ASIC2 could be regulated by miRNA in a rs28936 allele-specific way.
We did not analyze other variants on 3’UTR of ASIC2 simply because our starting hypothesis was to give a biological meaning to a statistically significant association between rs28936 and MS, and also because we have previously studied other variants, which resulted not significant or less significant than rs28936.

EJN European Journal of Neuroscience FENS Federation of European Neuroscience Societies

Anyway, our choice does not exclude the possibility that other variants located on 3'UTR could be possible miRNA binding sites and consequently could be investigated in further studies also by other research groups.
We also analyzed the association between rs28936 and MS in UK MS brain tissue, on which we have studied ASIC2 mRNA, and we now report this analysis in the paper. The result of the association was β=1.02[-0.02; 2.21], p=0.068, marginally significant with upper confidence interval shifted toward values higher than zero. This borderline significance is probably due to the small sample size.
The reason why we limited our study on mRNA and miRNA of ASIC2 and not of ASIC1 was because the main aim of this paper was driven by prior hypothesis concerning ASIC2. To study ASIC1 mRNA and miRNA regulation, for us could certainly be an interesting topic for grant application or/and for other research group the hint for further investigation.
We added a specific paragraph in material and methods named "Workflow of the study", in which we specify the logic followed to plan our experiments.

2. For the two types of behavioral experiment, the clinical signs of EAE, and the mechanical allodynia, it is critical that the researchers carrying out the experiment are blinded to the genotype of the animal. There is however no mention of this in the Methods. The authors need to state in the methods whether the experimenters were blinded to the mouse genotype or not.
We have now reported in the methods section that the behavioral experiments were performed by an unbiased method carried out by an expert experimenter aware of the mouse genotype.

3. The mechanical allodynia experiments showed a higher threshold for ASIC2(-/-) mice than others under control conditions, and a lowering of the threshold by EAE in all three genotypes. There is no evidence provided for a statistically different effect of EAE on mechanical allodynia between genotypes. In the discussion (upper part of p.14) this observation is discussed as if the EAE-induced lowering of the threshold for mechanical allodynia was different in ASIC2(-/-) mice as compared to WT. This is however not shown. In the discussion of the increased threshold in ASIC2(-/-) mice under control conditions (discussion on lower part of p13), the authors should also mention studies that have carried out the same type of experiments (von Frey filaments on hind paw) and did not observe any difference between ASIC2(-/-) and WT, as for example Staniland et al., Eur J Pain (2009), and they should discuss possible reasons for this difference.
We agree with the reviewer and we modified the discussion accordingly and added the suggested reference.

Specific points:

1. The final statement of the abstract is too strong; the data provide only a weak support for a role of ASIC2 in MS.
We modified the last statement of the abstract.

2. P3, center of page, "ASIC2….involved in neurodegeneration in C. elegans…". This statement is wrong, degenerins, not ASIC2a are involved in neurodegeneration in C. elegans.
We corrected this statement in the introduction.

3. P5, lower part of first paragraph, "…showed that 4-aminopyridine, …inhibits heterotrimeric ASIC channels". This information should not be pushed too much as an evidence for an involvement of ASICs in MS, since clinical concentrations of 4-aminopyridine reached in MS therapy are below 1 microM, and the IC50 for ASIC inhibition by 4-aminopyridine is close to 1 mM.
After a thorough consideration we agree with the reviewer and we decided to eliminate this sentence, also in an effort to simply the introduction as suggested by the same reviewer at point 8.

4. P12, top paragraph, analysis of rs28936 genotype link to MS; this analysis should be shown.
We added this analysis in the results section (paragraph "ASIC2 mRNA expression is increased in MS brain").

5. P15, upper half, "The induction of has-miR-27a…" This statement and much of this paragraph needs to be declared more clearly as a hypothesis/speculation.
We agree with the reviewer and we changed this statement and other parts in the paragraph accordingly.

6. P15, bottom, "The increased pain thresholds in mice … suggests a possible role of ASIC2a in the pathophysiology of MS…". It is not correct to use results of the mechanical allodynia as an argument for an involvement of ASIC2 in EAE or MS. The study in its present form does not show evidence for a difference between ASIC2(-/-) mice and WT in EAE-induced pain.
We agree with the reviewer and we changed the sentence accordingly.

7. **Table 1, title of the 4th column from left, "AA construct, Dicer.." should probably be "GG construct, Dicer.."**
Yes. We have corrected our error.

8. The introduction and discussion are very long and should be shortened.
We have shortened both the introduction and the discussion, also clarifying our research hypothesis.

Reviewer #3:

Major points

The results shown in figure 1 can be somewhat confusing, after reading it several times you understand what they mean, what really confuses me is that the authors refer the maximum clinical score peak for Asic1- / and Asic2- / - on day 27 and 25 respectively, but checking those days in the graph do not seem

EJN European Journal of Neuroscience FENS Federation of European Neuroscience Societies

to coincide with the maximum values, for example in the case of Asic2 - / - I notice the maximum value in the clinical assessment on day 36.
We apologize for this. We now report the correct maximal clinical score peaks in the results section.

Minor points

Page 2 row 4. Specify that "rs28936" refers a Single Nucleotide Polymorphisms.
We have specified that "rs28936" refers a Single Nucleotide Polymorphisms.

Page 4 row 2. They talk about the role of ASIC2a but they never mention the characteristics and functions of the ASIC2b subunit.
On Page 4 (middle page) we have now mentioned the characteristics and functions of the ASIC2b subunit.

Page 5 row 7. Why do they use lowercase letters in "Asic1"?
Page 11 row 19. Why do they use lowercase letters in "Asic1"?
In both cases we have corrected in the text the use of ASIC1 lowercase.

Page 11 row 12. The significant increase of CD4+ in the Asic2$^{-/-}$ mice is not mentioned
Page 13 row 15. They do not discuss the increase of CD4+ that is observed in the infiltrate present in Asic2 - / - mice.
We now report that the increase of CD4^{+} mononuclear cells in Asic2$^{-/-}$ mice in the results and discussion sections.

Page 15 row 24. Specify "The increased pain thresholds in Asic2-/- mice"
We have changed this sentence accordingly to the comment.

Figure 2 is not very readable, I recommend increasing its size.
We uploaded a new figure.

2nd Editorial Decision 08-Nov-18

Dear Prof. Bernardinelli,

Your revised manuscript was re-evaluated by external reviewers as well as by the Section Editor, Dr. Yoland Smith and ourselves. Although the three reviewers consider that you have satisfactorily addressed most of their concerns, a critical issue remains in the study design. The fact that the behavioral analyses were not achieved in a blinded fashion is a serious concern that goes against the level of rigor and reproducibility expected in EJN publications. You must better validate this choice of not blinding the observers to the mouse genotype and possibly raise this as a major limitation in the discussion of your paper. Alternatively, if you

have data that were gathered in a blinded fashion that could corroborate your current findings, these should be included in the paper, to help address this important issue. Editorial re-review of the revised manuscript will be needed.

Additional queries are indicated in the comments below.

When revising the manuscript, please embolden or underline major changes to the text so they are easily identifiable and DO NOT leave 'track change' formatting marks in your paper. At this stage, please provide text and a figure file for the Graphical Abstract. When carrying out your revisions please refer to the checklist below and visit the EJN author guidelines at www.ejneuroscience.org

When finalised, please upload your complete revised manuscript onto the website as a Word (.doc or .docx), or .rtf file. Please also ensure that a complete set of tables and figures is included as separate files, even if these have not changed from the originals. At this stage it is necessary to provide high resolution figures. Please see important instructions below.

Please go into https://mc.manuscriptcentral.com/ejn - Author Centre - manuscripts with decisions where you will find a 'create a revision' link under 'actions'. We ask that you please indicate the way in which you have responded to the points raised by the Editors and Reviewers in a letter. Please upload this response letter as a separate Word (.doc) file using the file designation "Authors' Response to Reviewers" when uploading your manuscript files. Please DO NOT submit your revised manuscript as a new one. Also, please note that only the Author who submitted the original version of the manuscript should submit a revised version.

If you are able to respond fully to the points raised, we shall be pleased to receive a revision of your paper within 12 weeks.

Thank you for submitting your work to EJN.

Kind regards,

John Foxe & Paul Bolam
co-Editors in Chief, EJN

Reviews:

Reviewer: 2

Comments to the Author
The authors indicate in the revised manuscript that the experimenter of the behavioral studies with mice (clinical evaluation of neuronal signs in EAE, assessment of mechanical allodynia), was not blinded to the genotype of the mouse under study. To my understanding, it is standard practice to have a blinded experimenter in such types of behavioral experiments. In my view, the results loose in this case much of their value. The journal has to decide whether it accepts results from such analyses that were obtained by an experimenter who knew the mouse genotype. I check “minor revision” as recommendation, but consider it critical that the editor decides whether to accept these mouse data or not.
For the rest, the authors have clarified the logic underlying their study. The analysis of the association

between rs28936 and MS in the brain samples remains somewhat unsatisfactory, since it is not possible to confirm here this association that was found in the previous study. There are still some language issues that can be corrected by the authors or editorial team.

Reviewer: 3

Comments to the Author
After carefully reading I found that authors have significantly improved the manuscript, following all the review comments. I consider the manuscript has much more coherence and adequate presentation, so I recommend its publication.

Reviewer: 1

Comments to the Author
(There are no comments.)

Authors' Response 12-Nov-18

Dear Editor, Dear Reviewers,

Please, find enclosed the revised version of manuscript number EJN-2018-07-25771, prepared in the light of EJN Author's information & Check-list.

We are very glad to have satisfactorily addressed most of the Editor's and Reviewer's concerns.

As to the critical issue concerning the blinded behavioral analyses, we apologize with both the Editor and the Reviewer, that despite the experiments were carried out by an experimenter unaware (blinded) of mouse genotype, it was mistakenly reported "aware" in the manuscript. This has now been corrected in our final version of the manuscript.

We apologize again for the inconvenience caused.

Best regards,

Prof. Luisa Bernardinelli
